# Ethnobotanical Insights: Qualitative Analysis of Medicinal Plants in Colón Putumayo for Traditional Knowledge Preservation

**DOI:** 10.3390/plants12193390

**Published:** 2023-09-26

**Authors:** Maira Alejandra Bastidas-Bacca, Dayana Dayve-Bacca-Descance, Adriana del Socorro Guerra-Acosta, Erika Perea-Morera, Lucia Ana Díaz-Ariza, Diana López-Álvarez, Ana Milena Osorio-García

**Affiliations:** 1 Grupo de Investigación de Recursos Naturales Amazónicos, Faculty of Engineering and Basic Sciences, Instituto Tecnológico del Putumayo, Colón 861040, Colombia; mabastidas17s@itp.edu.co (M.A.B.-B.); aguerra@itp.edu.co (A.d.S.G.-A.); 2 Faculty of Engineering and Basic Sciences, Instituto Tecnológico del Putumayo, Colón 861040, Colombia; dayanadescanse@gmail.com; 3 Grupo de Investigación en Agroecología, Department of Biological Sciences, Universidad Nacional de Colombia—Sede Palmira, Palmira 763533, Colombia; epeream@unal.edu.co; 4 Grupo de Investigación en Agricultura Biológica, Biology Department, Sede Bogotá, Pontificia Universidad Javeriana, Bogotá DC 110231, Colombia; luciaana@javeriana.edu.co; 5 Grupo de Investigación en Diversidad Biológica, Department of Biological Sciences, Universidad Nacional de Colombia—Sede Palmira, Palmira 763533, Colombia; dilopezal@unal.edu.co

**Keywords:** traditional medicine, community health, alternative treatment of diseases

## Abstract

The ancestral knowledge of the community of Colón Putumayo unfolds in several dimensions that allow us to recognise the ethnomedicinal properties of plants. The research focused on systematising ethnobotanical and ethnomedicinal knowledge on the use of plants as alternatives for treating illnesses. A cross-sectional study was carried out through semi-structured questionnaires to 100 inhabitants of the community of Colón. We found 38 plant species catalogued in 18 botanical families where 10 species of medicinal plants were prioritised by the community for the treatment of illnesses. The use value (UV) evaluation showed that *Chamaemelum nobile* equals 0.18 compared to *Cymbopogon citratus* and *Lippia alba* with 0.04. The Informant Consensus Factor (ICF) for the cited medicinal use categories equivalent to 1.00 are for the treatment of six types of ailments, however, the plants can treat 16 types of ailments. The Fidelity Level (LF) found identified that four plants are used for the treatment of one type of ailment while three are used to alleviate several ailments. The local knowledge of the community of Colón Putumayo is linked to the ancestry of the territory, culture, and family farming practices.

## 1. Introduction

Traditional medicine, rooted in the ancient wisdom of diverse cultures, has long been recognized as a valuable source of healthcare practices and remedies [1]. Since immemorial times, it has represented a profound connection between human societies and the natural world, harnessing the therapeutic properties of plants and other natural resources [2]. This discipline known as ethnobotany explores the dynamic relationship between plants and people, with a particular focus on the traditional knowledge and practices of indigenous and local communities regarding plant uses. It encompasses the study of how different cultures perceive, interact with, and utilize plants for various purposes, including medicinal, culinary, spiritual, and cultural applications [3,4,5,6]. Ethnobotanical research seeks to understand plant-human interactions’ ecological, cultural, and social dimensions [5].

For decades, research has been conducted to explore the exciting world of traditional medicine, illuminating its effectiveness, cultural relevance, and even its influence on modern healthcare structures [7,8]. The World Health Organization (WHO) is aware of the potential benefits of traditional medicine and has formulated a plan for its integration into global health services [8]. Moreover, other studies have drawn attention to the connection between biological diversity and sustainability; underlining the interdependence between traditional medicine, biodiversity, and cultural heritage [1].

A review by Smith (2020) highlights the significance of information systems in preserving and utilizing traditional knowledge [9]. They emphasize the need for robust systems that can support the documentation, organization, and retrieval of ethnobotanical information to preserve not only the traditional knowledge as an invaluable resource for understanding and utilizing medicinal plants but can also contribute to discovering novel plant-based drugs [10,11] and support sustainable healthcare practices [12,13,14]. Furthermore, as traditional medicine and the associated use of medicinal plants is deeply rooted in cultural practices and beliefs, the preservation of traditional knowledge promotes cultural resilience, conserves their cultural heritage, and empowerment of the communities [15,16].

Latin America possesses a rich tradition of traditional medicine and ethnobotanical practices. Ethnopharmacological studies have been conducted to explore the diverse range of medicinal plants used in the region, unravelling their traditional uses and potential pharmacological properties [17]. These studies highlight the importance of documenting and preserving the valuable knowledge of traditional herbal remedies, with a focus on benefiting future generations [18,19].

Investigations into medicinal plants have illuminated their therapeutic properties, traditional meaning, and potential uses in modern medicine [20,21]. Calixto (2005) conducted an extensive review of medicinal plants in Latin America focusing on their customary functions, chemical structure, and pharmacology to underscore their cultural relevance and prospective therapeutic applications [22]. Additionally, Lopez (2011) investigated the ethnobotanical aspects of medicinal plants, calling attention to their cultural worth, traditional know-how systems, and the need for sustainable practices for conservation [23]. Moreover, Bennett and Prance (2000) conducted investigations on the indigenous pharmacopoeia of northern South America, revealing the incorporation of introduced plants into traditional healing practices [24]. Together, these studies contribute to an enhanced understanding of traditional medicine in Latin America, encompassing its cultural heritage, therapeutic potential, and the significance of preserving and sustainably utilizing medicinal plants for the well-being of present and future generations.

Colombia is a country known renowned for its remarkable biodiversity, which includes a wide range of ecosystems that harbour an abundance of plant species known for their medicinal properties. For generations, these plants have played a pivotal role in healthcare practices, benefiting Indigenous communities and the general population alike [25]. They have been utilized to address a wide range of conditions, preserving overall well-being, and serving as a testament to the profound traditional knowledge and cultural heritage surrounding medicinal plant use in Colombia [26]. Despite the country’s abundant natural resources, scientific understanding of its flora remains limited in several aspects [27]. Nevertheless, notable research conducted in Colombia has contributed significant insights into traditional medicine practices. Akerele (1993) emphasized the importance of preserving medicinal plants as a valuable resource with the potential to address global healthcare challenges, while Acevedo’s recent review (2020) offers a comprehensive overview of the current state of the field and underscores the need for further exploration [28,29].

In recent years, Colombia has made substantial progress in various domains and communities concerning the utilization of medicinal plants. Several relevant ethnobotanical surveys have been conducted in Colombia [30,31,32,33,34,35], documenting traditional healers’ traditional knowledge and utilization of medicinal plants across different regions. De Páscoa and de Souza (2021) undertook a systematic review of the utilization of medicinal plants used in the Amazonian region [36]; they aimed to compile and analyse existing knowledge to gain insight into the traditional use, therapeutic potential, and cultural significance of these plants in the region. These studies correlate with previous work done by Gonzalez (1980) and Schultes (2007), documenting and analysing the use of medicinal plants by traditional healers in this biodiverse and culturally rich area working together with indigenous communities, these studies, among others, highlight the wealth of traditional knowledge, cultural significance, and therapeutic potential of medicinal plants in Colombia [37,38]. They provide valuable resources for researchers, healthcare professionals, and policymakers seeking to promote the sustainable use of medicinal plants, preserve traditional knowledge, and integrate traditional medicine into modern healthcare systems.

The department of Putumayo is situated in the southern part of Colombia, within the Amazon region. This region, known as Amazonia, is a vital and extraordinary ecosystem of immense importance. It is admired for its remarkable biodiversity and the abundance of medicinal plants it harbours [39]. The Amazonia region is recognized for its distinct flora and the traditional knowledge passed down through generations regarding the use of these medicinal plants. Through scientific research and ethnobotanical surveys, the significance of the Amazonia region has been illuminated, underscoring its critical role in healthcare practices and the preservation of traditional knowledge [17,40]. The Sibundoy Valley, situated in Alto Putumayo within the Andean region, is one of Colombia’s most biodiverse areas [41,42]. Within this region’s rich ecological diversity, the Inga, Kamentzá, and Quillacinga ethnic groups coexist, well-known for their profound knowledge and extensive utilization of the plants found in their environment [41]. The Valley has gained recognition as a global hotspot for cultivated magical plants, serving as a significant repository of ancestral wisdom related to medicine and botany [43]. The Putumayo region, especially the Sibundoy Valley is well known for numerous studies about healing medicine such as Yage or Ayahuasca (medicine made from the *Banisteriopsis caapi* vine and other plant ingredients) [44,45,46,47,48,49,50,51,52,53,54]. Nonetheless, numerous researchers also have done ethnobotanical research exploring other medicinal plants used by this community. These studies investigated a variety of topics related to medicinal plants, such as traditional wisdom, alternative ethnobotany, data systems, and community-based approaches. All the research conducted here has had an essential effect on our knowledge of the medicinal vegetation in this region and its link with the local people [41,55,56,57,58,59].

Gallego-Pérez et al. (2021) revealed the importance of traditional medicine to indigenous cultures in Putumayo [26]. They urged for harmony between traditional and modern healthcare. Similarly, Vandebroek (2013) recommended community involvement in healthcare initiatives [60]. Anwar, (2010) argues that information systems can help preserve and disseminate traditional knowledge in an area. These studies have furthered our understanding of medicinal plants and traditional medicine [61]. González and Kröger (2020) highlighted the value of information systems for enlarging the accessibility and usability of traditional knowledge within Putumayo communities [62]. Nassar et al. (2020) proposed a collaborative approach that allows the documenting and safeguarding of traditional knowledge, based on investigations conducted the in Colombian Amazon [63]. Palacios Bucheli and Bokelmann, (2017) mentioned that integrating traditional knowledge and practices is crucial for the well-being of local communities, addressing the specific needs of local communities, focused on designing and developing an ethnobotanical knowledge management system [64,65]. Lastly, Rahman (2000) provided an information systems perspective on the digital documentation and analysis of traditional knowledge [66]. Together, these studies highlight the importance of integrating traditional knowledge, engaging local communities, and implementing effective information systems to preserve and utilize ethnobotanical information in the Putumayo region. This study aims to elucidate the traditional knowledge about the use of medicinal plants as ethnobotanical alternatives among the communities of Colón Putumayo, considering their curative potential and their importance for the local culture.

## 2. Results and Discussion

### 2.1. Socio-Demographic Characteristics

A total of 100 Colonense individuals including 10 males and 90 females with a mean age of 55.1 ± 15.6 (Table 1), were interviewed from 9 neighbourhoods and three veredas of which the majority 78% were ≥41 years old as compared to 22% aged ≤40 years (youth). Garzón-Garzón’s investigations in 2016 refer to the inhabitants of the indigenous reservation of MAC Amazonas, where the knowledge of medicinal plants predominates among the elderly, as opposed to young people who lack application and uses of medicinal plants [67]. On the other hand, in Brazil, research by Alves et al. (2016) mentions that approximately 80% of the Brazilian population uses, has used medicinal plants in their daily lives, where the majority are adults over 60 years of age [68]. In a study carried out by Liu et al. (2023), they mention that people over 40 years of age use medicinal plants first to combat symptoms of various diseases, in addition, people over 60 years of age are more satisfied with the efficacy of herbal medicine [69]. Additionally, 64% reside in urban areas and 36% in rural areas. Garzón-Garzón’s study revealed that 40% were women primarily engaged in domestic duties as housewives, while 39% were involved in diverse occupations such as fieldwork, repair activities, construction, and more; the remaining percentage was distributed among various other professions (Table 1). We reported more women participants due to their role in conserving indigenous knowledge of plants; Nalumansi et al. (2014) also found that 80% of interviewees were women because they knew of traditional medicine to meet their own healthcare needs and those of their children [70]. According to Torres-Avilez et al. (2016) the women in Latin American countries, generally, have more knowledge of medicinal plants than men [71], although this finding is not consistent across all studies, even some studies indicate that it is women who have traditional knowledge of medicinal plants related to improve women’s health [72].

### 2.2. Botanical Diversity and Habit

In this study, local communities of Colón Putumayo mentioned 38 species of medicinal plants, belonging to 34 genera and 18 families, that are used by local communities to treat diseases (Table 2). Lamiaceae and Asteraceae with 27%, and 19% species were the dominant medicinally utilized plant families, respectively (Table 3). However, Apiaceae, Amaranthaceae, Malvaceae, Poaceae, and Verbenaceae presented 5.3% of species each. The results are maintained according to research carried out by Toscano in 2006 where the families of plants most used by inhabitants of the department of Boyacá, Colombia were Asteraceae, Lamiaceae, and other families [73,74], on the other hand, in the north coast of Colombia, it is reported that the medicinal plants most used by the inhabitants were Asteraceae, Lamiaceae, Anacardiaceae, Annonaceae, Bignoniaceae, Cucurbitaceae, Euphorbiaceae, Liliaceae and Myrtaceae [75]. Likewise, in the Andean region of Colombia, the medicinal plants reported in the literature belong to four families: Asteraceae, Lamiaceae, Apiaceae, and Solanaceae [76]. In general, the species were classified under the least concern category of the International Union for Conservation of Nature (IUCN) list (Table 3). Another important aspect that should be highlighted is that 85% of the interviewees access these medicinal plants through cultivation in home gardens and traditional chagras.

**Figure 1:**
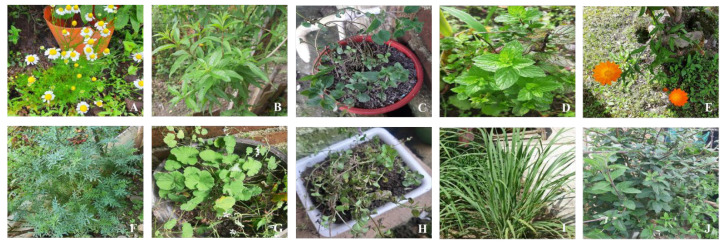
Medicinal plants most used by the community of Colón Putumayo. (**A**). *Chamaemelum nobile*; (**B**). *Lippia citriodora*; (**C**). *Origanum vulgare*; (**D**). *Mentha piperita*; (**E**). *Calendula officinalis*; (**F**). *Ruda graveolens*; (**G**). *Malva sylvestris*; (**H**). *Mentha polegium*; (**I**) *Cymbopogon citratus*; (**J**). *Lippia alba*.

Among them, ten plant species were identified as being the most frequently used (Figure 1). These include *Chamaemelum nobile* (chamomile) with a usage percentage of 18%, followed by *Lippia citriodora* (lemon verbena) with 17%, *Origanum vulgare* (oregano) with 16%, *Mentha piperita* (peppermint) with 12%, *Calendula officinalis* (marigold) with 9%, *Ruta graveolens* (rue) with 9%, *Malva sylvestris* (common mallow) with 6%, *Mentha pulegium* (pennyroyal) with 5%, *Cymbopogon citratus* (lemongrass) with 4%, and *Lippia alba* (Mexican oregano) with 4%, similar results were reported by Rodriguez, (2020) (Table 3) [41].

The medicinal plants recorded possess different growth (habit) forms such as grass (63.2%), small shrub (28.9%), trees (2.6%), and liana (2.6%), where 24 of them are cultivated, four are native, four are naturalized, two are adventitia, one is introduced and one is naturalized adventitia, for two medicinal plants no information is available (Table 3).

### 2.3. Parts used for Medicine and Methods of Preparation

The medicinal plants that prevail in use are intended for the treatment of seventeen causes of diseases that correspond to the ethnomedicinal knowledge identified by the community of Colón (Table 3). It was possible to identify that the main uses of the plants are aimed at the treatment of infectious or parasitic diseases, diseases of the digestive system, of the circulatory system, and diseases related to problems of the genitourinary system similar results were found by Arias (2003) [110].

In Latin America, the use of medicinal plants is closely related to magical, culinary, craft and religious uses [111]. While countries like China, Korea, India, Indonesia, Malaysia, Myanmar, Sri Lanka, Thailand, and Vietnam, among others, focus on the pharmaco-logical uses of medicinal plants [112]. This investigation showed that the use of these plants was focused on implementing their consumption to prevent or mitigate the incidence of diseases or to avoid and/or delay the appearance of sequelae or chronic diseases, according to the worldview of the Colón Putumayo community; the practices of use and management of plants are closely related to the cosmogony of these communities and the knowledge of natural cycles [41,57]. But in this case it was not possible to demonstrate the use of these plants for magical, spiritual or religious purposes, as has been the case of *Ruda graveolens* and *Petiveria alliacea*, which have been reported in other studies as amulets, baths, incense, bouquets or drinks [41].

**Figure 2:**
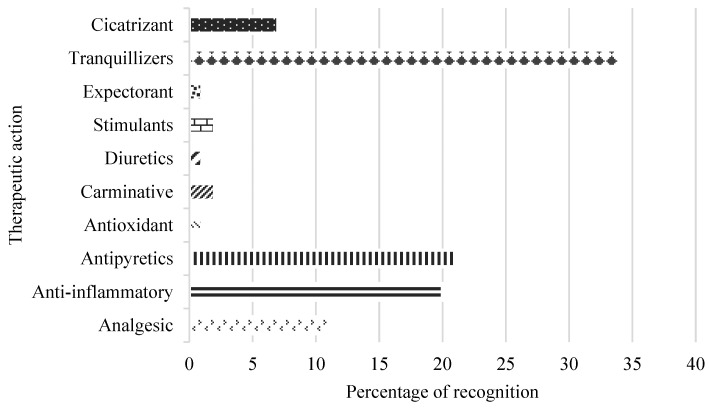
Therapeutic actions attributed to medicinal plants.

The plants were used as a treatment for various diseases, at least it was evidenced that a medicinal plant has the potential to treat five causes of disease or symptoms. The local population attributes medicinal plants to therapeutic actions such as tranquilizers, antipyretics, anti-inflammatory, analgesic, and healing, mainly (Figure 2).

The infusion is the main way to prepare medicinal plants to extract their benefits, followed by the decoction −17%- and the poultice −1%- there are also forms of application such as baths and ointments. The main part of the plant used for the preparation is the leaves and to a lesser extent the flowers or the stem in the fresh state (Table 3).

### 2.4. The Use-Value (UV)

The highest use value reported in this research was 0.18 and 0.17 for *Chamaemelum nobile* and *Aloysia citrodora* respectively, while the lowest was 0.04 (Table 4) for *Cymbopogon citratus* and *Lippia alba*. The high use value in plants could be attributed to their wide distribution and diverse application uses, making them the first choice for treatment. The present study attempts to address quantitative ethnobotanical data on disease treatment in the community of Colón Putumayo.

### 2.5. Informant Consensus and Categories

The Informant Consensus Factor (ICF) identified a strong consensus (>0.90) for 16 categories found, with a greater emphasis on six categories (ICF = 1) as follows: blood, blood forming organs and immune system, digestive system diseases, social problems, urinary system, nervous system diseases, pregnancy, and childbearing (Table 5).

### 2.6. Fidelity Level (FL)

FL was determined for the plants most used by the informants to treat a particular category of disease [113]. Values equivalent to one hundred percent of FL indicate that all informants (in a specific category) use the plant to treat only that category of disease [113]. FL less values of 100% indicate that the plant was used in other diseases [114]. Table 6 shows the FL values for the ten plants prioritized for the study frequently in each disease category. The highest FL values (100%) were identified in four plants, where *C. officinalis* is used for skin problems, *C. citratus* is used for the treatment of coughs and to relieve the symptoms of tuberculosis, *L. alba* for muscle relaxant and for abdominal pain and finally, *R. graveolens* for the treatment of menstrual cramps.

The species used for various types of ailments are *A. citrodora*, *M. sylvestris* and *M. spicata* with values corresponding to 11%, 10% and 9% respectively.

### 2.7. Quantitative Factors

Based on the quantitative indices, the UV values were determined in the plants used in traditional medicine in Colón Putumayo; The highest UV values were calculated for *Chamaemelum nobile* (0.18); according to the ICF results for the 10 ICPC disease categories and the 16 categories for the international standard for Primary Care. The analysed data showed that few plants were cited by most of the informants for their medicinal value.

The community of Colón has cultural roots and endogenous ancestral knowledge that has allowed the ethnomedicinal knowledge of its territory to be transcended and transmitted, through sovereignty, land tenure ―95% owned and 5% leased―, family farming practices and access to plant material for planting. When inquiring with the inhabitants, 81% indicate that their knowledge about medicinal plants is maintained by tradition and because a relative taught it to them, while 16% affirm that they have studied or read about the subject. It was possible to show that the community of Colón Putumayo has valuable ethnobotanical knowledge that has transcended ancestral wisdom and that has also been built over time in the territory.

The community of Colón uses a diversity of medicinal plants for the treatment of illness or diseases, as an alternative to alleviate symptoms given that the Colombian medical system takes time to attend to in a timely manner, especially in rural communities. The forms of preparation, the applied use and the frequency are linked to the knowledge acquired from generation to generation or by the tradition of the community of Colón. Of the 38 species of plants used in the municipality of Colón, 24 grow in grassy habitats that are easily accessible and cultivated (Table 3).

Three forms of preparation preferred by the community are used: in-fusion, decoction, and poultice, however there are other methods of preparation such as syrups, creams, capsules and other types depending on the treatment and the symptoms that each person presents.

Medicinal plants are mainly used to treat ailments of the digestive system, genital system, general diagnoses, and diseases. The species *C. nobile* and *A. citrodora* have high use values for the population of Colón, where they are used for specific treatments such as stomach pain and digestive problems, and for relaxation, tranquillity and nerves-related issues, respectively.

## 3. Materials and Methods

### 3.1. Study Area

**Figure 3:**
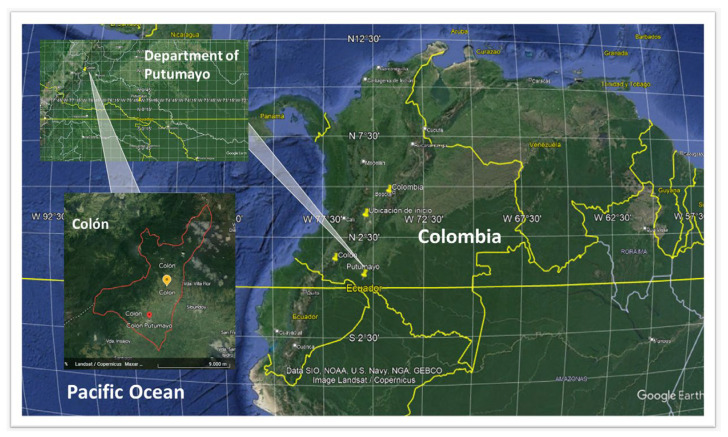
The geographical location of the municipality of Colón, Putumayo. Coordinates: Lat. 1 1.1830556; Long. −76.96694444444445. Image edited from Google Earth, 2023 ©Copyright Google Earth 2009 [118].

The research was conducted in the municipality of Colón, located in the Putumayo department in the Colombian Andes, specifically in the subregion of the Sibundoy Valley (Figure 3). Colón is situated in Alto Putumayo and is one of the four municipalities that make up the Sibundoy Valley. It is situated at an altitude ranging from 2100 to 3500 m above sea level. The municipality covers an area of approximately 75.38 Km^2^, with an average temperature of 16 °C [41]. The climate varies between 8 °C in winter and 21 °C in summer, with an average annual rainfall of 1580 mm [115]. The average annual relative humidity is 80%, and the area falls within the life zones of Lower Montane Very Wet Forest (Bmh-MB) and Montane Wet Forest (Bh-M) [116]. The municipality has a population of around 5358 inhabitants, with 2238 residing in rural areas and 3120 in urban areas [117].

### 3.2. Ethnobotanical Data Collection and Plant Identification

Between March and April 2022 were conducted semi-structured interviews for a total of 100 participants from nine neighbourhoods and three veredas (rural areas corresponding to the administrative divisions of a municipality); all research participants were volunteers and gave their permission to be part of the study through oral consent as required by the International Society of Ethnobiology [119]. The names and addresses of participants were excluded to maintain confidentiality. Interviewees were considered ranging in age from 23 to 83 years old, and they recognize themselves from different communities: rural Colonense populations, indigenous communities, Quillacinga and Pasto peoples, as well as a settlement of Afro-descendants who possess knowledge of and utilize medicinal plants; among them are individuals who practice herbal medicine, including traditional healers, shamans, and curanderos.

The interviews were focused on considering different aspects, such as knowledge, uses, preparation methods, consumption practices, and specific plant parts employed with relevant ethnographic information (Table 1). The field work was done by visiting each chagra (it comes from the Quechua language which means farm. It is an area arranged by the indigenous communities to cultivate); Chagras differ in size, but in general, they had a size of less than 5.000 m^2^ (half a hectare), but some of them had small backyards. These are heterogeneous places where a variety of plants are cultivated for different purposes, that provide the family with food and medicine, allowing them to have food sovereignty. Therefore, the interviews considered only the plants that they had grown in their gardens, orchards, or yards, due to their importance. Following data collection in the field, the specimens of medicinal plants underwent botanical identification, were identified using the botanical taxonomy method and were contrasted with the herbarium voucher; and a literature review was conducted to complement, validate, and cross-reference the ethnomedicinal knowledge associated with these plants. The scientific plant names were given according to the Plant List www.theplantlist.org (accessed on 13 September 2023) and International Plant Name Index www.ipni.org (accessed on 13 September 2023) databases.

### 3.3. Data Analysis

Data were analysed using descriptive (reports, frequencies, and percentages) and three quantitative ethnobotanical methods: use value (UV), informant consensus factor (ICF), and level of fidelity (FL) [120]

The use-value (UV) shows the relative importance of species and was calculated with the following formula:

UV = UVi/Ni (1)

where “UVi” is the number of citations for species across all interviewees and “Ni” is the number of interviewees [121].

The Informant Consensus Factor (ICF), is used as an indicator of the level of homogeneity of the ethnomedicinal information of a plant species [122]

ICF = (Nur − Nt)/(Nur − 1) (2)

where Nur = the number of use reports from informants for a particular plant use category and Nt = the number of taxa or species that use the category for all informants. The ICF values range from 0 to 1. Low ICF values (close to 0) indicate that interviewees disagree about the usage of the plant for a particular ailment category, while high ICF values (near 1) indicate that a limited number of plant species are cited by a higher proportion of interviewees to treat a specific disease.

Finally, the fidelity level (FL), is used to determine the plants most used by interviewees in a disease category [113] using the following formula:

FL = (Np/N) × 100 (3)

where Np is the number of interviewees who cited or mentioned the use of a medicinal plant for a particular disease category and N is the total number of interviewees who cited that plant for any other use or purpose [123,124]; FL high value indicates that a medicinal plant has the highest use report and the most preferred species within a particular disease category. We used 16 different use or disease categories adapted and modified from the International Classification of Diseases 11th (ICD-11) Revision of the World Health Organization [125].

## 4. Conclusions

Our expedition into the realm of traditional knowledge and ethnobotanical alternatives in Colón Putumayo communities has unveiled an abundant collection of medicinal plants brimming with extraordinary healing properties. Through this qualitative analysis and exploration of their cultural significance, we have unearthed insights that complement the existing literature on the subject.

Traditional medicine serves as a vital nexus between human societies and the bountiful natural environments that surround them. Delving into diverse cultural practices and awareness the remarkable medicinal properties of plants, traditional medicine offers a comprehensive approach to healthcare, while setting the stage for a more focused exploration of Colombia’s contributions to this ancient field.

The study managed to carry out an analysis of the ethnobotanical uses of medicinal plants according to their use value, the consensus values of the residents of Colón Putumayo according to the types of ailments which revolve around the treatment of blood, blood-forming organs, and immune system; digestive system diseases; nervous system diseases; pregnancy and childbearing and, urinary system.

There are four species that are used by the community of Colón Putumayo for the treatment of a disease, while three medicinal plants are used to treat various types of diseases such as *Mentha spicata* for the treatment of stress, *Aloysia citrodora* used as a tranquillizer and *Malva sylvestris* for stomach pains and conditions in the throat.

## Figures and Tables

**Table 1:** Socio-demographic characteristics of participants.

Variables	Categories	N. of Informants in Each Category (%)
Sex ratio	Female	90
	Male	10
Age groups	Between 20 and 40 years	22
	Between 41 and 60 years	42
	Above 60 years	36
Educational level	Primary level	77
	Secondary level	34
	Technical studies	15
Occupation	Farmer	9
	Housewife	40
	Trader	5
	Herbal healer	2
	Public Employee	1
	Nurse	2
	Rancher	2
	Miscellaneous trades	39
Life type	Urban areas	64
	Rural areas	36

**Table 2:** Main medicinal and ethnomedicinal plants used by the Colón Putumayo community to treat diseases.

Common Name	Scientific Name	Ethnomedicinal Uses	International Classification of Primary Care	Medicinal Uses of Plants According to Causes of Disease	Reference
Chamomile	*Chamaemelum nobile*	Curative properties for the treatment of problems related to the skin and hair. They attribute properties to cure dental and stomach pain and digestive problems, prevent vomiting, and facilitate the expulsion of intestinal gases. On the other hand, chamomile is used as a mouthwash.	Digestive system Genital system Eye General diagnoses and diseases Skin	Digestive system diseases: used to treat stomach cramps. Diseases of the genitourinary system: treatment of menstrual pain, kidney, and bladder disorders. Symptoms, signs, or clinical results related to the visual apparatus: treatment of minor infections and inflammation in the eyes. Skin diseases: treatment of superficial wounds.	[77,78,79]
Kidron	*Aloysia citrodora*	It is used mainly as a relaxant, tranquillizer, and for the nerves, it has also been used to treat anxiety and digestive disorders. The community attributes antioxidant compounds to it.	Social problems Musculoskeletal system General diagnoses and diseases Digestive system diseases Blood, blood-forming organs, and immune system Urinary system Genital system Endocrine, metabolic, and nutritional system	Diseases of the nervous system: use as a calming, sedative, and nervous tranquillizer. Diseases of the musculoskeletal system or connective tissue: use as an antispasmodic. Infectious or parasitic diseases: use as an expectorant, treatment of colds and flu. Digestive system diseases: used to relieve abdominal pain and gastrointestinal spasms, improves intestinal constipation. Diseases of the circulatory system: use for arterial hypertension. Diseases of the genitourinary system: use as a diuretic. Symptoms, signs, or clinical findings not elsewhere classified: Antipyretic treatment and headaches. Endocrine, nutritional, or metabolic diseases: appetite stimulant.	[80,81,82,83,84,85]
Oregano	*Origanum vulgare*	The Colón Putumayo community uses oregano to treat stomach aches or pain related to menstruation. They also warn that the infusion should be taken for a maximum of six weeks in a row and recommend avoiding consumption for pregnant and lactating women.	Psychological, mental, and neurodevelopmental Digestive system Skin General diagnoses and diseases Endocrine, metabolic, and nutritional system General symptoms, complaints, and abnormal findings Genital system Circulatory system Musculoskeletal system	Diseases of the nervous system: it has sedative properties. Digestive system diseases: Antidiarrheal, promotes digestion, and is antispasmodic. Skin diseases: treatment of skin infections and as a repellent. Infectious or parasitic diseases: Antifungal, antibacterial, treatment of respiratory diseases, laryngitis, and for treatment of malaria, likewise, it produces bronchoalveolar secretions. Endocrine, nutritional, or metabolic diseases: treatment of liver disorders and diabetes. Symptoms, signs, or clinical findings not classified elsewhere in the body: Analgesic, antipyretic, anti-inflammatory, and sedative. Diseases of the genitourinary system: use as a diuretic, for menstrual disorders. Diseases of the circulatory system: Antihypertensive. Diseases of the musculoskeletal system or connective tissue: Antispasmodic, relieves torticollis and lumbago. Conditions related to sexual health: treatment of syphilis and gonorrhoea. Pregnancy ending in abortion: abortive.	[86,87]
Peppermint/Good herb	*Mentha spicata*	Peppermint is used to relieve stress and anxiety. On the other hand, they consume mint to relieve nausea and vomiting.	Digestive system General diagnoses and diseases Skin Circulatory system Genital system	Digestive system diseases: Antispasmodic, favour the expulsion of gases from the digestive system, improves symptoms of constipation, and colitis and helps control digestive disorders. Some infectious or parasitic diseases: Anti-inflammatory and decongestant of the respiratory system, antiseptic on the mucous membranes, and flu-like symptoms. Skin diseases: Antiseptics on the skin. Diseases of the circulatory system: cardiovascular and anti-hypochondriac decline. Diseases of the genitourinary system: Anti-dysmenorrhea and antispasmodic. Diseases or disorders of the orofacial complex: Anti-inflammatory and analgesic on the teeth.	[88,89,90,91]
Calendula	*Calendula officinalis*	The people of Colón maintain that the marigold has attributes to reduce inflammation and heal wounds or skin lesions. They acknowledge that the plant can cause drowsiness and slow breathing if combined with sedative medications.	Skin Genital system Genital system General diagnoses and diseases Musculoskeletal system Genital system Endocrine, metabolic, and nutritional system Psychological, mental, and neurodevelopmental Psychological, mental, and neurodevelopmental Circulatory system	Skin diseases: Anti-inflammatory, healing, and diaphoretic action. Use for treatment of dermatitis, wound healing, and skin eruptions. Diseases of the genitourinary system: diuretic. Digestive system diseases: Antispasmodic, treatment of digestive tract and gastritis. Infectious or parasitic diseases: control of parasitic diseases, bacterial, fungal, and viral diseases. Use for the treatment of pharyngitis. Diseases of the musculoskeletal system or connective tissue: use in bruises. Diseases of the genitourinary system: treatment of amenorrhea and dysmenorrhea. Endocrine, nutritional, or metabolic diseases: treatment for liver failure and hepatitis. Stimulate bile secretion. Diseases of the nervous system: migraines. Symptoms, signs, or clinical findings not classified elsewhere in the body: Analgesic, antipyretic, anti-inflammatory, and sedative. Diseases of the circulatory system: treatment of hypertension, tachycardia, and arrhythmia. Nervous system diseases: tranquillizer.	[92,93,94,95,96]
Rue	*Ruta graveolens*	The most frequent use is for the treatment of menstrual cramps, stimulants of menstrual flow, and abdominal cramps. Another use was for the treatment of intestinal parasites. They also mention that rue is used to treat “bad hour” or “bad wind”, that is, for the treatment of various diseases such as headaches, rheumatism, diabetes, and sterility. Also, having bad wind is related to the treatment of the evil eye which is related to weakness in the spirit or from birth. Rude is also used to treat skin damage.	Nervous system diseases Digestive system Pregnancy and childbearing Digestive system Psychological, mental, and neurodevelopmental Musculoskeletal system Skin General diagnoses and diseases Eye	Diseases of the circulatory system increase the resistance of blood capillaries, treatment for heart and vascular problems, and nosebleeds, and are used for the treatment of varicose veins. Diseases of the genitourinary system: treatment of amenorrhea, dysmenorrhea, menstrual pain, and uterine bleeding. Pregnancy ending in abortion: abortive. Diseases of the digestive system: treatment of digestive disorders and haemorrhoids. Nervous system diseases: nervous tranquillizer. Some infectious or parasitic diseases: treatment for respiratory conditions and tuberculosis. Symptoms, signs, or clinical findings not elsewhere classified: treatment for headache. Diseases of the musculoskeletal system or connective tissue: treatment for rheumatism, dropsy, and gout. Skin diseases: treatment for insect bites, exanthema, abscesses, psoriasis, and skin damage. Some infectious or parasitic diseases: treatment for cough, scabies, and pediculosis. Diseases of the visual system: conjunctivitis. Diseases or disorders of the orofacial complex: treatment of ulcers on the gums.	[97,98,99,100]
Scented mallow	*Malva sylvestris*	It is used for the treatment of stomach pains and conditions in the throat. It is also used as an aromatic to accompany the meals of the day.	Digestive system Psychological, mental, and neurodevelopmental General diagnoses and diseases Eye Circulatory system	Digestive system diseases: treatment of digestive problems, haemorrhoids and used as a laxative. Skin diseases: helps healing, and treatment of wounds, sores, ulcers, and insect bites. Diseases of the nervous system: use as a sedative. Some infectious or parasitic diseases: used as an expectorant, used for the treatment of the respiratory system, cough, colds, pharyngitis, and bronchitis. Symptoms, signs, or clinical findings not classified elsewhere in the body: used as analgesics and anti-inflammatories for various pains. Diseases or disorders of the orofacial complex: treatment of ulcers on the gums and oral lesions. Diseases of the visual system: eye care. Symptoms, signs, or clinical findings not classified elsewhere in the body: relieve itching and used as an anti-inflammatory. Diseases of the circulatory system: chest pain.	[101]
Pennyroyal	*Mentha pulegium*	It is commonly used for the treatment of stomach pains and colic.	Digestive system General diagnoses and diseases Musculoskeletal system Genital system	Symptoms and signs related to food and liquid intake: used for loss of appetite and anorexia. Digestive system diseases: treatment for constipation, gastrointestinal spasms, bile problems, diarrhoea, flatulence, indigestion, nausea, vomiting, and meteorism. Symptoms, signs, or clinical findings not classified elsewhere in the body: treatment for headache. Diseases of the musculoskeletal system or connective tissue: treatment of pain, muscle cramps, and rheumatism. Some infectious or parasitic diseases: treatment for respiratory congestion, cough, bronchitis, mucous and throat problems. Diseases or disorders of the orofacial complex: Anti-inflammatory and analgesic on the teeth. Diseases of the genitourinary system: treatment to relieve menstrual pain.	[102,103,104]
Lemongrass	*Cymbopogon citratus*	The community uses lemongrass to treat coughs and to relieve the symptoms of tuberculosis.	Psychological, mental, and neurodevelopmental General diagnoses and diseases General symptoms, complaints, and abnormal findings General diagnoses and diseases Digestive system	Mental, behavioural, and neurodevelopmental disorders: Antidepressant use. Some infectious or parasitic diseases: used to relieve congestion, asthma, cough, and respiratory problems. Neoplasms: treatment to inhibit the growth of cancer cells. Symptoms, signs, or clinical findings not classified elsewhere in the body: Analgesic, antipyretic, anti-inflammatory, and sedative. Digestive system diseases: treatment for flatulence, digestive problems, antispasmodic and carminative.	[105,106]
Soon relief	*Lippia alba*	Prompt relief is mainly used as an essential oil for the treatment of various body pains. The community stated that it is used as a muscle relaxant and for abdominal pain.	Digestive system Genital system General diagnoses and diseases Psychological, mental, and neurodevelopmental Skin Endocrine, metabolic, and nutritional system	Digestive system diseases: treatment for digestive problems, haemorrhoids, and colic. Used as a laxative, antispasmodic and carminative. Diseases of the genitourinary system: use as a diuretic. Some infectious or parasitic diseases: use as an expectorant, antibacterial, or antifungal. Used to treat flu, and cough. Sleep and wakefulness disorders: use as a sleeping pill Diseases of the nervous system: use as a sedative and anticonvulsant. Skin diseases: promote the secretion of sweat. Used for the treatment of skin ulcers and healing. Symptoms, signs, or clinical findings not classified elsewhere in the body: treatment for fever. Diseases of the genitourinary system: treatment for hypermenorrhoea. Endocrine, nutritional, or metabolic diseases: treatment for liver failure and hepatitis.	[107,108,109]

**Table 3:** Identification of medicinal plants used, methods of preparation, states of the plant, and parts used in the treatment of diseases by the community of Colón Putumayo.

Family	Scientific Name	Common Name	Common Local Name	Biogeography	Habit	IUCN Category Red List
*Amaranthaceae*	*Alternanthera lanceolata*	Cancel	Descancel	No information	-	-
*Amaranthaceae*	*Chenopodium ambrosioides*	Paico	Paico	Naturalized	Grass	-
*Apiaceae*	*Anethum graveolens*	Dill	Eneldo	Cultivated	Grass	-
*Apiaceae*	*Apium graveolens*	Celery	Apio	Cultivated	Grass	LC
*Asteraceae*	*Artemisia abshintium*	Wormwood	Ajenjo	Cultivated	Bush	-
*Asteraceae*	*Chamaemelum nobile*	Chamomile	Manzanilla	Adventitia	Grass	LC
*Asteraceae*	*Bidens pilosa*	Pacunga	Pacunga	Adventitia	Grass	-
*Asteraceae*	*Calendula officinalis*	Calendula	Calendula	Cultivated	Grass	-
*Asteraceae*	*Cynara cardunculus var*	Artichoke	Alcachofa	Cultivated	Grass	LC
*Asteraceae*	*Taraxacum officinale*	Dandelion	Diente de león	Naturalized	Grass	LC
*Asteraceae*	*Munnozia hastifolia*	Arnica	Árnica	No information	Grass	-
*Caprifoliaceae*	*Valeriana officinalis*	Valerian	Valeriana	Cultivated	Grass	LC
*Equisetaceae*	*Equisetum bogotense*	Horse tail	Cola de caballo	Native	Grass	-
*Lamiaceae*	*Malva sylvestris*	Scented mallow	Malva olorosa	Cultivated	Bush	LC
*Lamiaceae*	*Salvia rosmarinus*	Rosmery	Romero	Cultivated	Bush	LC
*Lamiaceae*	*Thymus vulgaris*	Thyme	Tomillo	Cultivated	Bush	LC
*Lamiaceae*	*Origanum majorana*	Marjoram	Mejorana	Cultivated	Bush	-
*Lamiaceae*	*Origanum vulgare*	Oregano	Oregano	Cultivated	Grass	LC
*Lamiaceae*	*Mentha spicata*	Peppermint/good herb	Hierva buena	Cultivated	Grass	LC
*Lamiaceae*	*Mentha pulegium*	Pennyroyal	Poleo	Cultivated	Grass	LC
*Lamiaceae*	*Melissa officinalis*	Melissa	Toronjil	Cultivated	Grass	LC
*Lamiaceae*	*Ocimum basilicum*	Basil	Albahaca	Cultivated	Grass	-
*Lamiaceae*	*Mentha*	Peppermint	Menta	Cultivated	Grass	-
*Malphighiaceae*	*Basnisteriopsis caapi*	Chundur	Chundur	Cultivated	Liana	-
*Malvaceae*	*Althaea officinalis*	Marshmallow	Malvavisca	Cultivated	Bush	LC
*Malvaceae*	*Malva sylvestris*	Lying hollyhock	Malva tendida	Cultivated	Bush	LC
*Monimiaceae*	*Peumus boldus*	Plant acetaminophen	Acetaminofén planta	Introduced	Tree	LC
*Nyctaginaceae*	*Salpianthus purpurascens*	Sweet nitro	Nitro dulce	Native	Bush	-
*Oxalidaceae*	*Oxalis corniculata*	Chulco	Chulco	Naturalized	Grass	-
*Plantaginaceae*	*Plantago major*	Broadleaf plantain	Llantén	Naturalized	Grass	LC
*Poaceae*	*Megathyrsus maximus*	Little tiger	Tigresillo	Naturalized, adventitia	Grass	-
*Poaceae*	*Cymbopogon citratus*	Lemongrass	Limoncillo	Cultivated	Grass	-
*Rutaceae*	*Ruta graveolens*	Rue	Ruda	Cultivated	Bush	LC
*Solanaceae*	*Solanum nigrum*	Nightshade	Hierba mora	Native	Grass	-
*Urticaceae*	*Urtica urens*	Nettle	Ortiga	Cultivated	Grass	LC
*Verbenaceae*	*Aloysia citrodora*	Kidron	Cedrón	Cultivated	Bush	-
*Verbenaceae*	*Lippia alba*	Soon relief	Pronto alivio	Native	Bush	-
*Xanthorrhoeaceae*	*Aloe vera*	Sabila	Sabila	Cultivated	Grass	-

**Table 4:** The value of the use of medicinal plants by the community of Colón Putumayo.

Scientific Name	Number of Citations for Species	The Use-Value
*Chamaemelum nobile*	18	0.18
*Aloysia citrodora*	17	0.17
*Mentha spicata*	12	0.12
*Calendula officinalis*	9	0.09
*Ruta graveolens*	9	0.09
*Mentha pulegium*	5	0.05
*Cymbopogon citratus*	4	0.04
*Lippia alba*	4	0.04
*Origanum vulgare*	16	0.16
*Malva sylvestris*	6	0.06

**Table 5:** Informant consensus factor (ICF) for the categories of medicinal use cited by community interviewees of Colón, Putumayo.

N	Types of Ailments	No taxa of Plants Used. (Nt)	Number of Use Citations (Nur)	ICF Value
1	Blood, blood-forming organs, and immune system	1	17	1.00
2	Circulatory system	4	43	0.93
3	Digestive system	9	83	0.90
4	Digestive system diseases	1	17	1.00
5	Endocrine, metabolic, and nutritional system	4	46	0.93
6	Eye	3	33	0.94
7	General diagnoses and diseases	11	104	0.90
8	General symptoms, complaints, and abnormal findings	2	20	0.95
9	Genital system	9	99	0.92
10	Musculoskeletal system	5	56	0.93
11	Nervous system diseases	1	9	1.00
12	Pregnancy and childbearing	1	9	1.00
13	Psychological, mental, and neurodevelopmental	7	57	0.89
14	Skin	6	68	0.93
15	Social problems	1	17	1.00
16	Urinary system	1	17	1.00

**Table 6:** FL values for the most named medicinal plants in Colón Putumayo for each category.

Specie Used	Used Part of the Medicinal Plant	State of the Plant	Preparation Method	Popular Use	N	Np	FL%
*Origanum vulgare*	Leaves	Fresh and dry	Infusion	Stomach pain and digestive problems and treatment related to menstruation	40	18	45%
*Calendula officinalis*	Flower	Fresh and dry	Extract	Skin	2	2	100%
			Infusion				
			Ointment				
*Chamaemelum nobile*	Leaves and flower	Fresh	Infusion	Stomach pain and digestive problems	40	12	30%
			Decoction				
			Other forms				
*Cymbopogon citratus*	Flower and stems	Fresh	Infusion	Treat coughs and relieve the symptoms of tuberculosis	8	8	100%
*Lippia alba*	Leaves	Fresh	Infusion	Muscle relaxant and for abdominal pain	3	3	100%
			Oils				
			Plaster				
			Other forms				
*Aloysia citrodora*	Leaves, flowers, and stems	Dry	Infusion	Relaxant, tranquilizer and for the nerves	46	5	11%
*Malva sylvestris*	Leaves and flower	Fresh	Infusion	Stomach pains and conditions in the throat	40	4	10%
			Plaster				
			Other forms				
*Mentha spicata*	Leaves and stems	Fresh and dry	Extract	Stress and anxiety	46	4	9%
			Infusion				
*Mentha pulegium*	Leaves	Fresh	Infusion	Digestive system	40	16	40%
			Decoction				
*Ruta graveolens*	Leaves and stems	Fresh	Infusion	Treatment of menstrual cramps, stimulants of menstrual flow, and abdominal cramps	3	3	100%
			Decoction				
			Plaster				
			Ointment				
			Other forms				

## Data Availability

Not applicable.
